# Causal effect of immune cells, metabolites, cathepsins, and vitamin therapy in diabetic retinopathy: a Mendelian randomization and cross-sectional study

**DOI:** 10.3389/fimmu.2024.1443236

**Published:** 2024-10-01

**Authors:** Huijun Zhou, Jingzhi Wang, Xuehao Cui

**Affiliations:** ^1^ Department of Endocrinology, The Affiliated Yancheng First Hospital of Nanjing University Medical School, The First People's Hospital of Yancheng, Yancheng, Jiangsu, China; ^2^ Department of Radiotherapy Oncology, The Affiliated Yancheng First Hospital of Nanjing University Medical School, The First People’s Hospital of Yancheng, Yancheng, Jiangsu, China; ^3^ John Van Geest Centre for Brain Repair and MRC Mitochondrial Biology Unit, Department of Clinical Neurosciences, University of Cambridge, Cambridge, United Kingdom; ^4^ Cambridge Eye Unit, Addenbrooke’s Hospital, Cambridge University Hospitals, Cambridge, United Kingdom

**Keywords:** diabetic retinopathy, cathepsin, immune cell, metabolite, vitamin, Mendelian randomization, cross-sectional study, NHANES

## Abstract

**Background:**

Diabetic retinopathy (DR) is a major microvascular complication of diabetes and a leading cause of blindness worldwide. The pathogenesis of DR involves complex interactions between metabolic disturbances, immune cells, and proteolytic enzymes such as cathepsins (CATs). Despite various studies, the precise roles of different CATs, metabolites, and vitamins in DR remain unclear.

**Method:**

In this study, we employed Mendelian Randomization (MR) to assess causal relationships using genetic instruments selected based on genome-wide association studies (GWAS). We employed two-sample and mediation MR to explore the causal effects between nine CATs, immune cells, metabolites, vitamins, and DR. Additionally, the study also incorporated data from the NHANES survey to explore the associated relationship between vitamins and DR. We utilized cross-sectional data from the NHANES to analyze the association between vitamin intake and diabetic retinopathy (DR), adjusting for potential confounders to strengthen the validity of our findings.

**Results:**

The MR analysis identified CAT H as a significant risk factor for both NPDR and PDR, with no evidence of reverse causality. Additionally, 62 immune cell traits were found to have causal relationships with NPDR and 49 with PDR. Enrichment analysis revealed that metabolic pathways such as sphingolipid metabolism are crucial in DR progression. Vitamins B6 and E were significantly associated with a reduced risk of PDR. Cross-sectional data indicated that vitamins B1, B2, B6, B12, and E progressively decreased with DR severity.

**Conclusion:**

This study is the first to identify CAT H as a key risk factor for DR, while vitamins B6 and E showed significant protective effects, particularly against PDR. These findings suggest that CAT H, along with vitamins B6 and E, could serve as therapeutic targets for DR. Further validation through larger, multi-center studies is recommended to enhance the accuracy and applicability of these findings.

## Introduction

Diabetic retinopathy (DR) is the most common microvascular complication of diabetes and the leading cause of preventable blindness in the adult working population, affecting over 100 million people globally in 2020 (1–3). The Global Burden of Disease study identifies DR as the fifth leading cause of blindness and significant visual impairment in individuals aged 50 and older. Projections indicate that the global number of people affected by DR will increase to 129.84 million by 2030 and 160.5 million by 2045 (4). DR, characterized by neurovascular degeneration due to chronic hyperglycemia, affects 34.6% of diabetic patients globally (5, 6). It can be classified into non-proliferative DR (NPDR), and proliferative DR (PDR), with PDR posing a severe risk of complete vision loss (7). The incidence rates for PDR, diabetic macular edema, and vision-threatening DR are 7.0%, 6.8%, and 10.2% respectively, highlighting the urgent need for new preventive and therapeutic approaches (5). Despite advancements in DR management, optimal diagnostic indicators and therapeutic approaches are lacking (8). Current treatments, including laser photocoagulation, anti-VEGF drugs, and ocular steroids, have limitations: lasers can cause permanent retinal damage, anti-VEGF therapy risks endophthalmitis, and ocular steroids often increase intraocular pressure (9–11).

Cathepsins (CATs), a group of lysosomal proteolytic enzymes, are crucial for cellular homeostasis and are involved in numerous physiological and pathophysiological processes, including protein and lipid metabolism, autophagy, and lysosome-mediated cell death (12–15). Their critical roles in these processes make them significant in various diseases, including diabetes (16, 17). Recent studies have revealed the roles of several CATs, including CAT B (18), C (19), D (20), L (21), and S (22), in either promoting or suppressing diabetes and complications like diabetic cardiomyopathy, diabetic nephropathy, and diabetic kidney disease. However, only a limited number of studies and clinical trials have explored the association between cathepsins and DR. A previous study found that the downregulation of CAT B, D, and L is associated with high glucose-induced anti-autophagic and pro-apoptotic effects in retinal vascular endothelial cells, suggesting a novel pathogenic mechanism and potential therapeutic targets for PDR (23). Another study found that CAT G released by neutrophils, along with neutrophil elastase (NE) and proteinase 3, contributes to the development of DR by enhancing the inflammatory response (24). A study found that CAT H is implicated in several diseases, including high myopia, atherosclerosis, type 1 diabetes, neuroinflammation, and brain atrophy (25). Furthermore, recent studies have found that CATs interact complexly with immune cells. CATs C and H are expressed in cytotoxic T lymphocytes and natural killer cells, aiding in the activation of granzymes; CAT B and L help maintain adaptive immune response homeostasis by regulating T and B lymphocyte cell death (26). Some CATs have complex interactions with serum metabolites, influencing the development of various diseases, including diabetes (20). Additionally, some studies found that vitamins can regulate the expression and function of CATs, demonstrating therapeutic effects in certain diabetes-related diseases through this pathway (27, 28).

Previous studies indicate significant differences in CATs roles across diabetic complications, the causal relationships between different CATs and DR, as well as their interactions with immune cells and metabolites in DR, remain underexplored. Therefore, further investigation is needed to clarify the causal relationships between different types of CATs and the risk of DR, which could potentially reveal new therapeutic targets. With advancements in genomics, increasing evidence highlights the role of heritability in disease etiology. Mendelian randomization (MR), which uses genome-wide association studies (GWAS) to employ genetic variants as instrumental variables, can infer causal effects of exposure on outcomes; MR analyses have been used to investigate the causal effects of different cathepsins on the risk of lung cancer and its histological subtypes using both univariable and multivariable methods. In our study, we used multi-omics MR and mediation MR to explore the causal relationships between immune cells, blood metabolites, and CATs with NPDR and PDR, as well as the potential therapeutic roles of certain vitamins in PDR. Subsequently, by utilizing data from the National Health and Nutrition Examination Survey (NHANES), we investigated the association between these vitamins and NPDR and PDR, further validating their therapeutic roles.

## Methods and materials

### Study design of MR analysis

**Figure 1:**
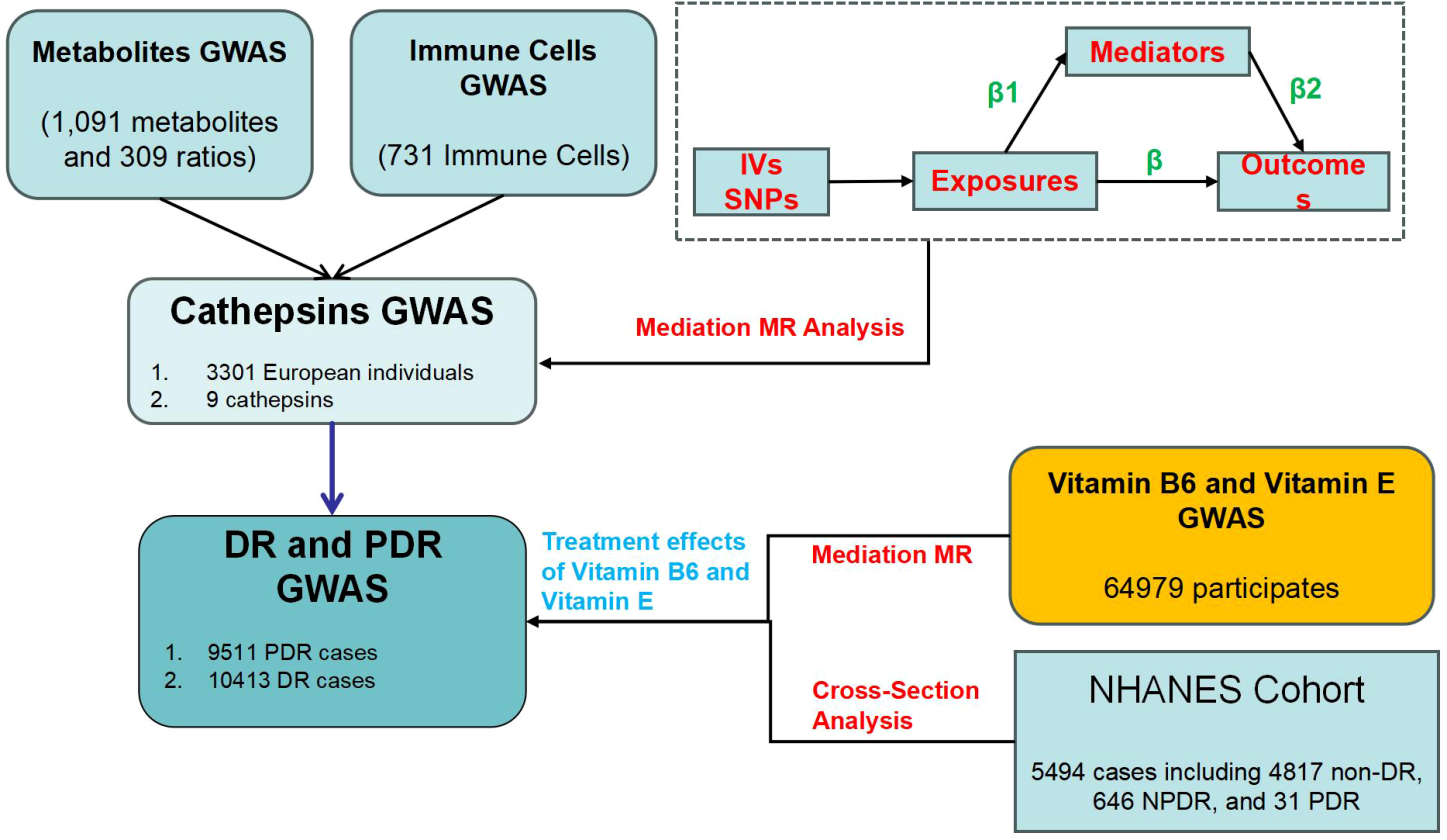
The study design and review.

Our MR study consists of three main steps (**Figure 1**). First, we performed a two-sample MR analysis to evaluate causal relationships between 9 CATs and DR, as well as between 731 immune cells/traits and 1400 metabolites/traits with DR. Second, we explored whether immune cells and metabolites mediate the causal relationship between CATs and DR, using enrichment analysis to investigate immune-metabolic mechanisms. Third, we examined the causal relationships between vitamins B6 and E and PDR, identifying their potential therapeutic roles and the mediating effect of CATs. To ensure the validity of our MR analysis, we met three key assumptions: (1) Genetic variants must be significantly associated with the exposure; (2) The selected instrumental variables (IVs) should not be correlated with confounders affecting both exposure and outcome; and (3) There should be no horizontal pleiotropy, meaning IVs should influence the outcome only through the exposure (29). The study employed two-step MR analyses to evaluate and quantify the mediating effects of selected mediators on the causal pathway between exposures and outcomes. In the first step, MR analysis determined the causal impact (β1) of exposures on each mediator. In the second step, MR analysis assessed the causal influence (β2) of each mediator on the outcome risk. The mediation proportion was calculated by dividing the product of the mediation effects (β1×β2) by the total effect between exposures and outcomes (30).

### Data sources of Mendelian randomization analysis

GWAS data for NPDR (GWAS ID: finngen_R9_H7_RETINOPATHYDIAB_BKG, 4,011 cases and 344,569 controls), PDR (GWAS ID: finngen_R9_DM_RETINA_PROLIF, 9511 cases and 362581 controls), were obtained from FinnGen (freeze 9) (31). This included 10,413 DR cases and 308,633 controls, 9,511 PDR cases and 362,581 controls. We identified cases of NPDR and PDR using International Classification of Diseases codes, specifically ICD-9 (3620) and ICD-10 (H360) for DR, and ICD-10 (H3603) for PDR.

The GWAS data on immune traits came from a study exploring the genetic basis of immune cell characteristics (32). 731 immune cell phenotypes were classified into four groups: absolute cell counts (AC), median fluorescence intensity (MFI), morphological parameters (MP), and relative cell counts (RC).

The GWAS dataset on metabolites was obtained from a study investigating the genetic determinants of metabolite profiles (33). Researchers examined 1,091 metabolites and 309 ratios, identifying genetic associations for 690 metabolites and 143 ratios across various loci.

Genetic instruments for assessing the levels of various CATs were sourced from the INTERVAL study, which included 3,301 European individuals (34). All participants provided informed consent, and the INTERVAL study was approved by the National Research Ethics Service (11/EE/0538). Summary data are available at https://gwas.mrcieu.ac.uk.

The GWAS data on vitamin B6 and vitamin E was derived from a substantial cohort of European ancestry within the UK Biobank (UKB). This dataset, compiled in 2018, included 64,979 participants and featured a comprehensive analysis of 9,851,867 single nucleotide polymorphisms (SNPs). This large-scale genetic evaluation provides a robust foundation for exploring the genetic determinants associated with vitamin B6 and E levels.

### Selection of genetic variants and MR analysis

No sample overlap was detected because the source populations of all the GWAS data included were diverse. We ensured that the selected SNPs were significantly associated with the exposures, with all SNPs linked to immune cells, and metabolites meeting the genome-wide significance threshold (P < 5 × 10^−8^). Additionally, we selected a separate set of SNPs below the locus-wide significance level (P < 5 × 10^-6^) as instrumental variables for CATs, this value was established in line with the limitation of the sample size. We performed linkage disequilibrium (LD) analysis with strict criteria (R² < 0.001, clumping distance = 10,000 kb) to adhere to MR assumptions. We performed this step using the *gwasvcf* package in R (35). Instrument strength was measured by the F-statistic, variance explained by r^2^, and proxy SNPs with r^2^ > 0.8 were used when exposure SNPs were absent from the outcome dataset (36). We used the LD matrix from the 1000 Genomes Project (European Utah residents) and included only results with at least three independent SNPs and an average F-statistic over 10 to ensure robust MR analyses (37). Genetic variants were harmonized by aligning the effect sizes (betas) to the same effect allele with the *TwoSampleMR* package (38). We utilized various analytical methods to validate our findings, the inverse variance-weighted (IVW) method was applied to estimate the overall impact of exposures on outcomes (39). Depending on the presence of heterogeneity, either a fixed or random effects model was used for the IVW analysis. When significant heterogeneity (P < 0.05) was detected, a random-effects IVW model was chosen (40). Effect sizes were reported using beta coefficients, odds ratios (OR), and 95% confidence intervals (CI).

### Sensitivity analysis

Sensitivity analyses were performed to ensure the robustness of the MR findings regarding the causal relationship between exposures and DR. Cochrane’s Q method was used to assess heterogeneity among the IVs, with P-values less than 0.05 indicating potential heterogeneity. Pleiotropic effects were initially evaluated using the intercept from MR-Egger regression, with P-values below 0.05 suggesting potential pleiotropy in the IVs. Additionally, the leave-one-out approach was implemented to assess the influence of individual SNPs, thus reinforcing the reliability of the MR analysis in establishing causality between exposures and DR.

### Enrichment analysis

We conducted an enrichment analysis on metabolites with significant effects (P < 0.05) on DR, aligning them with a curated reference of metabolic pathways. Enrichment ratios were calculated by dividing the number of metabolites within each pathway by the total number of metabolites listed in that pathway from the reference set. To determine the statistical significance of enrichment for each pathway, we used the hypergeometric test, which accounts for the sizes of both the reference set and the specific metabolite group.

### Data sources of cross−sectional study

The NHANES is an ongoing series of cross-sectional surveys targeting non-institutionalized civilians in the United States. Utilizing a multistage probability sampling technique, NHANES selects a sample that accurately represents the national population. It evaluates participants’ health and nutritional status through a combination of household interviews, physical examinations, and laboratory tests. This survey is managed by the National Center for Health Statistics (NCHS), part of the Centers for Disease Control and Prevention (CDC). Detailed information on the sampling method and data collection procedures is available in prior publications. All statistical analyses accounted for the NHANES complex survey design, incorporating sample weights, stratification, and clustering to ensure estimates are representative of the U.S. population. This methodology enhances the generalizability of the findings and reinforces the validity of the research in a population health context. The study received ethical approval from the NCHS Ethics Review Board, and all participants provided written informed consent (41).

The data for the cross-sectional study originated from the results of 2 cycles in the NHANES (2005–2008, www.cdc.gov/nchs/nhanes) and included demographic data, dietary data, laboratory test results, examinations, and questionnaire results. A total of 20,497 participants were included in the study. Among them, 14,793 were excluded due to a lack of DR-related records, and 210 were excluded due to missing dietary intake data for vitamin B6 and vitamin E. Consequently, a total of 5,494 individuals were included in this cross-sectional study. The assessment of retinal photographs, available on the NHANES website, was meticulously conducted by at least two experienced experts using a rigorous procedure for the diagnosis and classification of DR. In the NHANES surveys, participants were required to provide written informed consent before enrollment.

### Covariates in analysis

The regression models were adjusted for covariates previously linked to vitamin intake and DR, including age, gender, race, education level, and BMI. Other relevant indicators such as blood glucose (GLU) and HbA1C were also included.

### Statistical analyses

In our study, we analyzed clinical data using EmpowerStats software and logistic regression models. We statistically described the baseline characteristics of the study population by DR subgroups. Continuous variables were presented as means with standard deviations (SD) and analyzed using weighted linear regression models. To evaluate the association between vitamin intake and DR, we determined beta values and 95% confidence intervals through multivariate linear regression analysis. The multivariate analysis included three models: model 1 with no adjustments, model 2 adjusted for gender, age, and race, and model 3 adjusted for all covariates. Smoothed curve fits were adjusted for variables simultaneously. Statistical significance was defined as P < 0.05. To reduce data volatility, we employed a weighting approach.

## Result

### Causal effect of CATs with DR in MR

**Figure 2:**
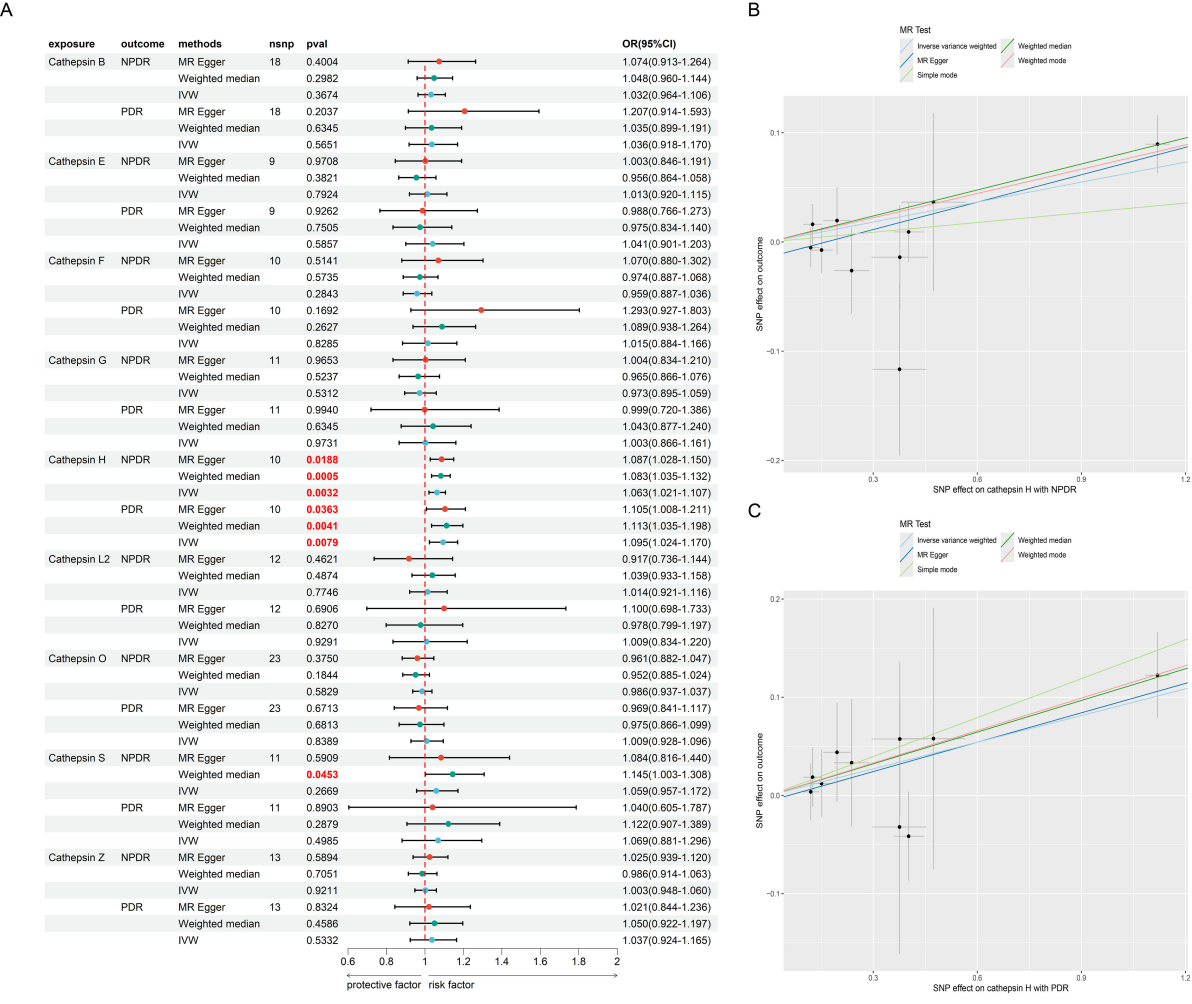
The MR result of cathepsins and DR. **(A)** the forest plot of MR result between cathepsins and DR. **(B)** the scatter plot of MR result between CAT H and NPDR. **(C)** the scatter plot of MR result between CAT H and PDR.

**Figure 3:**
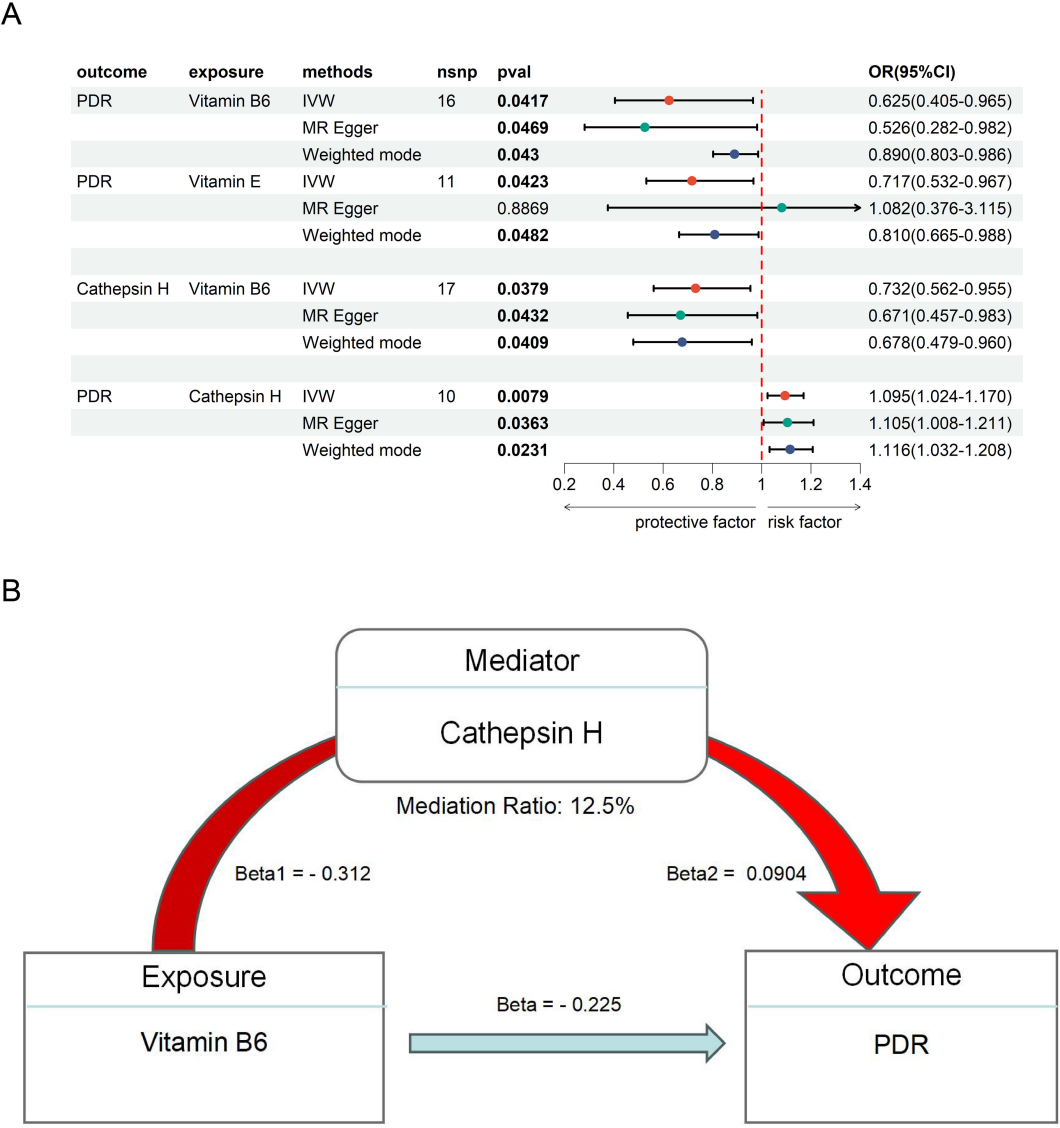
The MR result of vitamins and PDR. **(A)** the MR result of Vitamin B6, E and PDR. **(B)** the mediation effect of Vitamin B6, CAT H and PDR.

We first analyzed the causal relationship between 9 CATs (CAT B, E, F, G, H, L2, O, S, and Z) and DR (NPDR and PDR) using MR analysis. Of the 9 CATs, under the criterion of P<0.05, the IVW method found that CAT H was significantly associated with both NPDR and PDR. We conducted a false discovery rate (FDR) correction for our results to get the FDR-P. With an FDR < 0.05, we still found that CAT H has a significant causal relationship with NPDR (OR = 1.06, 95% CI 1.02 – 1.10, *P* = 0.0032) and PDR (OR = 1.09, 95% CI 1.02 – 1.17, *P* = 0.0079) (**Figures 2**, **3**). The causal results of CAT H with NPDR were found by MR analysis using other MR methods, including weighted median (OR = 1.08, 95% CI 1.03 – 1.13, *P* = 0.0005) and MR Egger (OR = 1.09, 95% CI 1.03 – 1.15, *P* = 0.019). Similarly, the results for CATs with PDR were doubly validated in both the weighted median (OR = 1.11, 95% CI = 1.04 – 1.20, *P* = 0.0041) and MR Egger (OR = 1.11, 95% CI = 1.01 – 1.21, *P* = 0.036) methods (**Figure 2**). We did not find heterogeneity and horizontal pleiotropy in these results (**Supplementary Table S1**). To explore the possibility of reverse causality, we conducted reverse MR analyses. These results indicated a lack of reverse causality between cathepsin H and the risk of NPDR and PDR (**Supplementary Table S2**).

### Causal effect of immune cells with DR in MR

**Figure 4:**
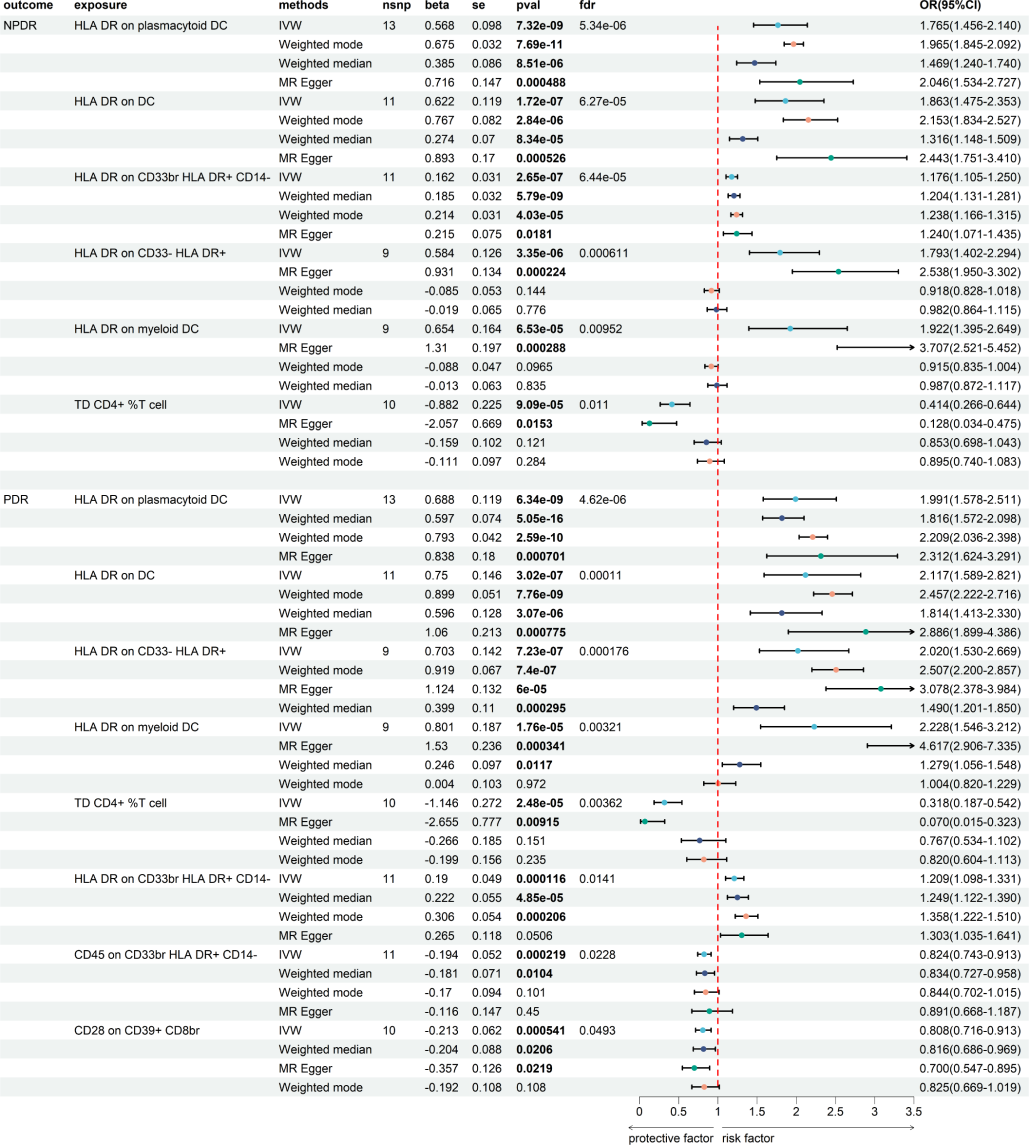
The MR result of immune cells and DR.

We then assess the causal effect between immune cells and NPDR and PDR (**Figure 4**; **Supplementary Tables S9, S10**). We found 62 immune cells/traits have a causal relationship to NPDR (*P* < 0.05) (**Supplementary Figure S1**) and 49 immune cells/traits have a causal relationship to PDR (*P* < 0.05) (**Supplementary Figure S2**). We applied the Benjamini-Hochberg (BH) method to adjust our results, obtaining enhanced FDR-adjusted P-values (FDR-P). We found that 6 immune cells/traits had FDR-P values less than 0.05 in NPDR and 8 immune cells/traits in PDR. The 6 immune cells with the most significant causal relationship to NPDR were as follows: HLA DR on plasmacytoid DC (beta = 0.568, *FDR-P* = 5.34E-06), HLA DR on DC (beta = 0.622, *FDR-P* = 6.27E-05), HLA DR on CD33br HLA DR+ CD14- (beta = 0.162, *FDR-P* = 6.44E-05), HLA DR on CD33- HLA DR+ (beta = 0.584, *FDR-P* = 0.00061), HLA DR on myeloid DC (beta = 0.654, *FDR-P* = 0.0095) and TD CD4+ %T cell (beta = - 0.882, *FDR-P* = 0.011) (**Figure 4**). The 8 immune cells with the most significant causal relationship to PDR were as follows: HLA DR on plasmacytoid DC (beta = 0.688, *FDR-P* = 4.62E-06), HLA DR on DC (beta = 0.750, *FDR-P* = 0.00011), HLA DR on CD33- HLA DR+ (beta = 0.703, *FDR-P* = 0.00018), HLA DR on myeloid DC (beta = 0.801, *FDR-P* = 0.0032), TD CD4+ %T cell (beta = - 1.146, *FDR-P* = 0.0036), HLA DR on CD33br HLA DR+ CD14- (beta = 0.190, *FDR-P* = 0.014), CD45 on CD33br HLA DR+ CD14- (beta = -0.194, *FDR-P* = 0.022) and CD28 on CD39+ CD8br (beta = - 0.213, *FDR-P* = 0.049) (**Figure 4**). We did not find heterogeneity and horizontal pleiotropy in these results (**Supplementary Tables S3, S4**).

### The causal effect between metabolites and the risk of DR and enrichment analysis

**Figure 5:**
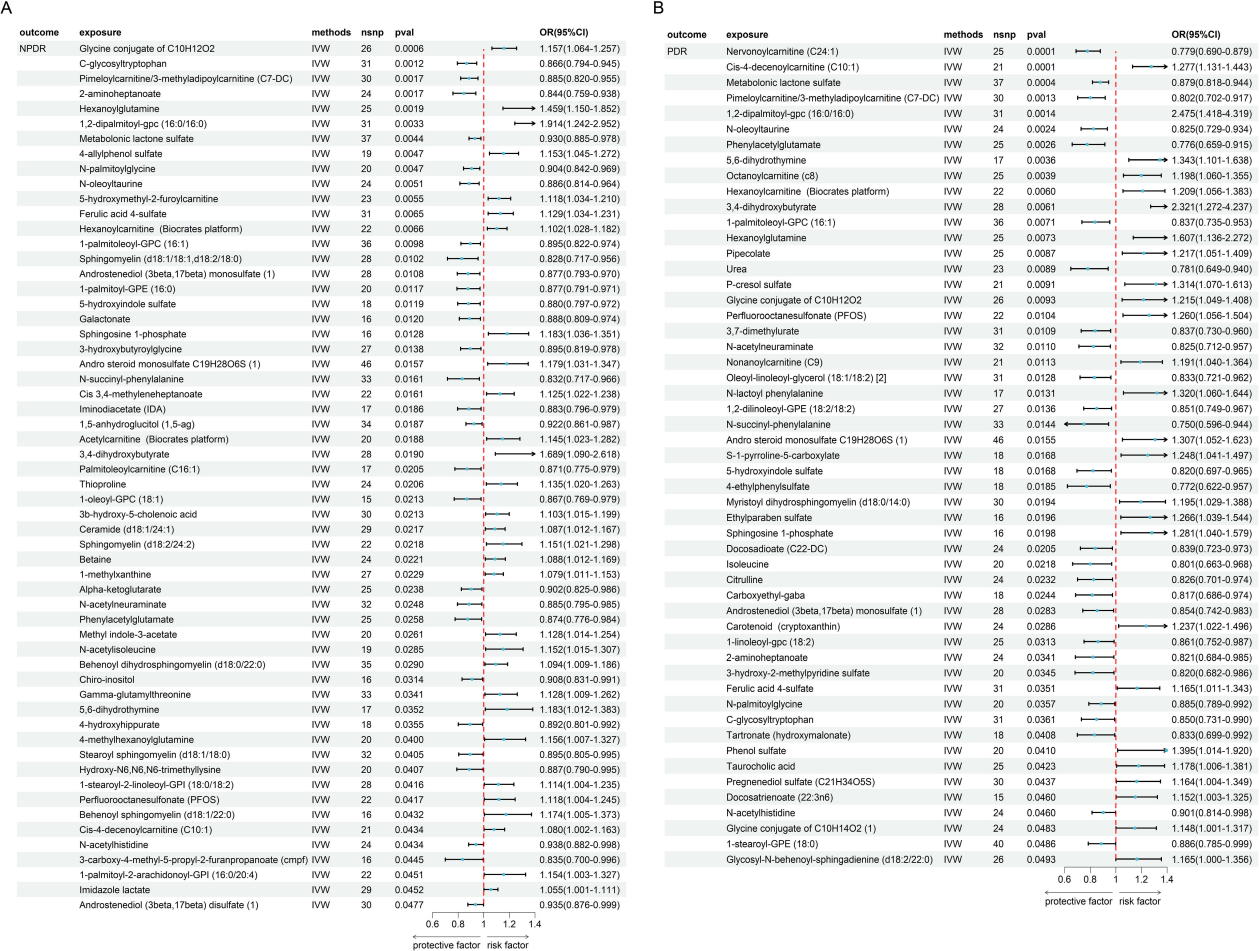
The MR result of metabolites and DR. **(A)** the result of metabolites and NPDR. **(B)** the result of metabolites and PDR.

We further investigated the causal link between metabolites and NPDR and PDR (**Supplementary Tables S11, S12**), as shown in **Figure 5**. This analysis identified 58 metabolites with a significant causal relationship with NPDR (*P < 0.05*, **Figure 5A**) and 53 metabolites with PDR (*P < 0.05*, **Figure 5B**). Among these, 30 metabolites were positively associated with an increased risk of NPDR, while 28 metabolites demonstrated a negative causal relationship, suggesting a protective effect against NPDR. In PDR, 26 metabolites were positively associated with an increased risk, while 27 metabolites demonstrated a negative causal relationship. We did not find heterogeneity and horizontal pleiotropy in these results (**Supplementary Tables S5, S6**).

**Figure 6:**
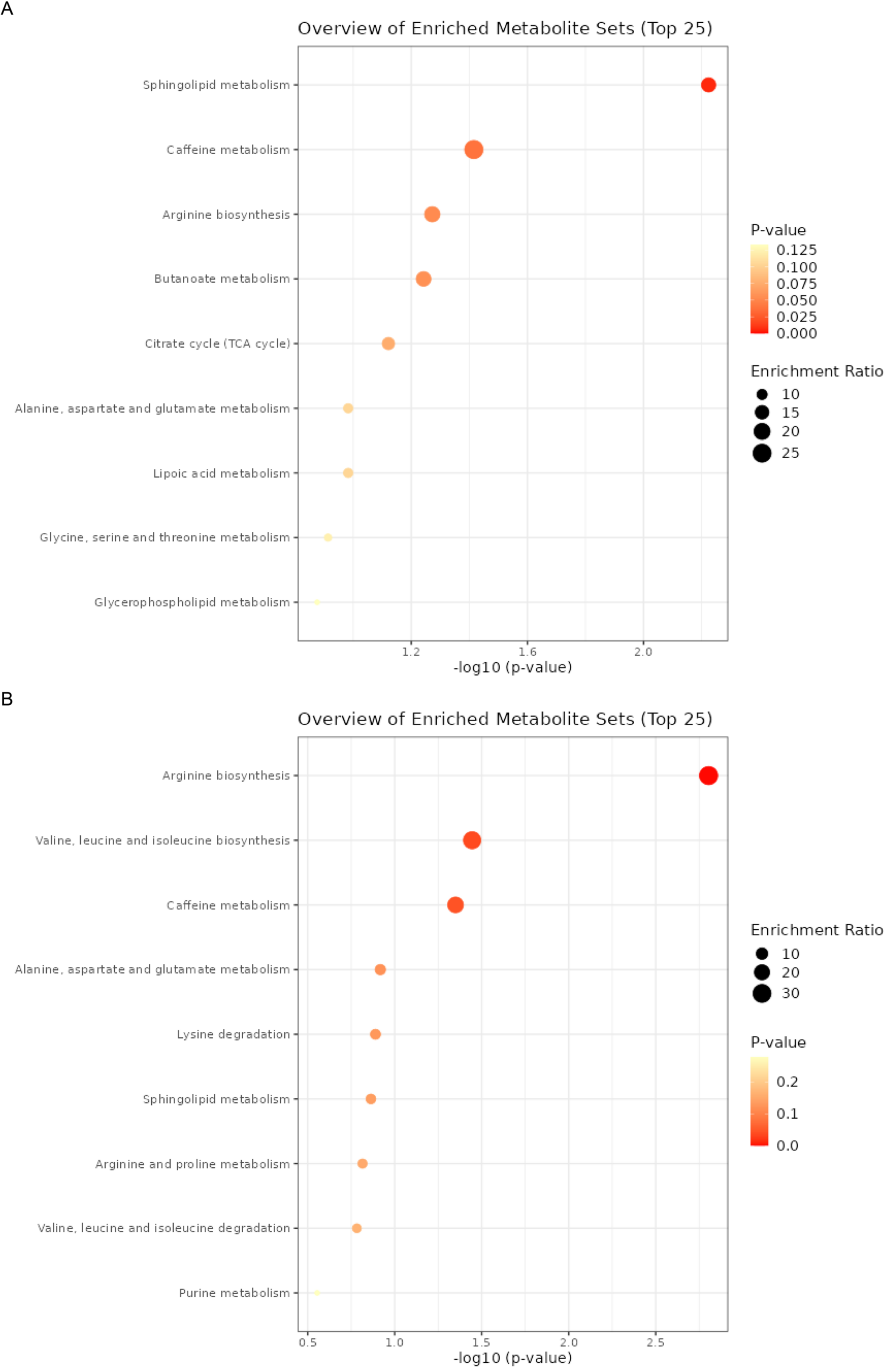
The enrichment analysis of metabolites. **(A)** the enrichment analysis of metabolites and NPDR. **(B)** the enrichment analysis of metabolites and PDR.

Subsequently, we conducted an enrichment analysis on these metabolites to identify potential metabolic pathways (**Figure 6**). In NPDR, the analysis revealed that Sphingolipid metabolism had the most significant impact, followed by pathways related to caffeine metabolism, arginine biosynthesis, and butanoate metabolism (**Figure 6A**). In PDR, the analysis revealed that arginine biosynthesis had the most significant impact, followed by pathways related to valine, leucine, and isoleucine biosynthesis, then caffeine metabolism (**Figure 6B**). We found that some pathways, such as caffeine metabolism, have a significant role in both NPDR and PDR. However, some pathways show considerable differences between the two groups. Since PDR develops from NPDR, we can infer that metabolic pathways significantly associated with PDR may be crucial in the progression of NPDR to PDR. Therefore, these pathways could potentially serve as important therapeutic targets for preventing and delaying the development of PDR.

### Immune cells mediate the causal effect of CAT H with DR

We used mediation MR analyses to explore the role of immune cells in the relationship between CAT H and DR (**Table 1**). Our findings indicated that HLA DR on CD33br HLA DR+ CD14- and HLA DR on CD14- CD16+ monocyte influences both NDPR and PDR via CAT H. Additionally, CD3 on NKT increased the risk of NPDR mediated by CAT H with a mediation proportion is 11.07%. The mediation effects of HLA DR on CD33br HLA DR+ CD14- and HLA DR on CD14- CD16+ monocytes in NPDR were 2.4% and 1.31%, respectively, and both were 3.04% in PDR.

**Table 1:** The mediation effect of Cathepsin H between Immune Cells and DR.

exposure	mediator	outcome	Step1		Step2		Total		Mediation Effect	Mediation Proportion
			Beta1	Pval	Beta2	Pval	Beta	Pval		
CD3 on NKT	cathepsin H	NPDR	0.178	0.012	0.0609	0.0032	0.098	0.047	0.0108	11.07%
HLA DR on CD33br HLA DR+ CD14-	cathepsin H	NPDR	0.0638	0.033	0.0609	0.0032	0.162	2.65E-07	0.00388	2.40%
HLA DR on CD14- CD16+ monocyte	cathepsin H	NPDR	0.0801	0.036	0.0609	0.0032	0.372	0.038	0.00488	1.31%
HLA DR on CD33br HLA DR+ CD14-	cathepsin H	PDR	0.0638	0.033	0.0904	0.0079	0.189	0.00012	0.00577	3.04%
HLA DR on CD14- CD16+ monocyte	cathepsin H	PDR	0.0801	0.036	0.0904	0.0079	0.238	0.037	0.00725	3.04%

### The potential therapeutic role of vitamins in DR

To further explore the therapeutics of DR, we conducted an MR analysis using Vitamins as the exposures and DR as the outcome. This analysis revealed a significant causal link between Vitamin B6 (OR = 0.625, 95% CI 0.405 - 0.965, *P* = 0.042), Vitamin E (OR = 0.717, 95% CI 0.532 - 0.967, *P* = 0.042), and PDR (**Figure 3A**), suggesting a potential protective or therapeutic effect of Vitamin B6, E against PDR. We did not find heterogeneity and horizontal pleiotropy in these results (**Supplementary Table S7**).

Next, we examined the mediating role of CAT H in this relationship by using Vitamin B6, and E as the exposures. Our analysis showed a significant negative causal relationship between Vitamin B6 and CAT H (OR = 0.732, 95% CI 0.562 - 0.955, *P* = 0.038), while CAT H was associated with an increased risk of PDR (OR = 1.10, 95% CI 1.02 - 1.17, *P* = 0.0079) (**Figure 3A**). These findings suggest that Vitamin B6 may protect against ON by influencing CAT H, with a mediating effect of 12.5% (**Figure 3B**).

### Baseline characteristics of population-based study from NHANES

In this investigation, there were a total of 677 DR patients and 4817 controls, with 547 participants classified as mild NPDR, 99 moderate/severe NPDR, and 31 confirmed as PDR (**Table 2**; **Supplementary Table S8**). The average age, gender distribution, race, and education levels of different groups are shown in **Table 2**. Building on prior MR studies, we assessed the relationship between vitamin B6, vitamin intake, and DR within this demographic. The average concentrations of vitamin B6 were measured at 74.6 mmol/L, 68.2 mmol/L, 55.3 mmol/L, and 49.0 mmol/L in non-DR, mild NPDR, moderate/severe NPDR, and PDR, respectively. Regarding vitamin intakes, significant differences were observed between the DR groups and the control group for vitamin B1, B2, B6, B12, and E, while differences in vitamin A, C, and K did not reach statistical significance. Specifically, the levels of vitamin B family and vitamin E were significantly lower in the DR group compared to the control group, with the lowest levels observed in the PDR group. The results from the NHANES study indicate that the severity of DR is significantly associated with the intake of B vitamins and vitamin E, the lower the intake, the more severe the DR. These findings are consistent with the previous MR analysis, suggesting that B vitamins and vitamin E could be important targets for the prevention or treatment of DR.

**Table 2:** Weighted characteristics of the study population based on DR.

Phenotype	Non-DR	NPDR		PDR	Pval
		Mild	Moderate/Severe		
N	4817	547	99	31	
AGE	59.209 ± 12.475	62.360 ± 12.039	61.677 ± 10.593	63.355 ± 7.405	**<0.001**
BMI	29.148 ± 6.467	29.739 ± 6.076	32.131 ± 6.993	31.724 ± 7.193	**<0.001**
GENDER					**0.001**
male	2375 (49.305%)	315 (57.587%)	51 (51.515%)	11 (35.484%)	
female	2442 (50.695%)	232 (42.413%)	48 (48.485%)	20 (64.516%)	
RACE					**<0.001**
Mexican American	739 (15.341%)	92 (16.819%)	25 (25.253%)	6 (19.355%)	
Other Hispanic	331 (6.871%)	44 (8.044%)	4 (4.040%)	5 (16.129%)	
Non-Hispanic White	2696 (55.968%)	253 (46.252%)	27 (27.273%)	5 (16.129%)	
Non-Hispanic Black	891 (18.497%)	142 (25.960%)	41 (41.414%)	15 (48.387%)	
Other Race	160 (3.322%)	16 (2.925%)	2 (2.020%)	0 (0.000%)	
EDUCATION					**<0.001**
< 9th Grade	644 (13.369%)	105 (19.196%)	20 (20.202%)	11 (35.484%)	
9-11th Grade	699 (14.511%)	99 (18.099%)	25 (25.253%)	6 (19.355%)	
High School Grad	1186 (24.621%)	141 (25.777%)	23 (23.232%)	5 (16.129%)	
College	1235 (25.638%)	137 (25.046%)	18 (18.182%)	7 (22.581%)	
College Graduate	1051 (21.819%)	65 (11.883%)	13 (13.131%)	2 (6.452%)	
**GLU**	5.719 ± 1.847	6.838 ± 3.330	10.059 ± 4.689	10.222 ± 5.466	**<0.001**
**HB1AC**	5.723 ± 0.894	6.381 ± 1.495	8.242 ± 1.999	8.023 ± 2.057	**<0.001**
**VITAMIN B6**	74.609 ± 91.447	68.187 ± 83.735	55.282 ± 56.540	48.997 ± 49.063	**<0.001**
Vitamin Intake					
Vitamin A	623.536 ± 766.767	623.380 ± 600.289	537.616 ± 588.045	633.065 ± 431.472	0.731
**Vitamin B1**	1.543 ± 0.868	1.460 ± 0.719	1.405 ± 0.843	1.274 ± 0.619	**0.021**
**Vitamin B2**	2.136 ± 1.204	1.996 ± 0.967	1.917 ± 1.270	1.832 ± 1.021	**0.009**
**Vitamin B6**	1.905 ± 1.180	1.674 ± 0.906	1.782 ± 1.436	1.446 ± 0.513	**<0.001**
**Vitamin B12**	5.349 ± 8.259	4.616 ± 5.173	4.515 ± 4.983	3.816 ± 2.899	**0.008**
Vitamin C	85.016 ± 87.144	79.025 ± 82.481	80.311 ± 95.975	101.468 ± 76.982	0.289
**Vitamin E**	7.119 ± 5.196	6.380 ± 4.190	6.593 ± 5.386	5.207 ± 2.990	**0.002**
Vitamin K	99.807 ± 157.349	99.930 ± 160.546	78.948 ± 82.121	156.916 ± 265.821	0.121

### Association between Vitamin B6, Vitamin E, and DR

In both previous MR and cross-sectional studies, vitamins B6 and E were identified as having a protective effect against DR. **Table 3** displays the outcomes from the multivariate regression analysis. In the unadjusted model, vitamin B6 [-0.023 (-0.033 ~ -0.013), *P* < 0.00001] and vitamin E [-0.004 (-0.007 ~ -0.002), *P* < 0.00032] were strongly associated with DR. Following adjustments for age, gender, race in model 2 and all covariates in model 3, the relationship between vitamin B6, E and DR were still significant (**Table 3**). These findings are consistent with the results from previous MR and Cross-Sectional studies, further confirming that vitamins B6 and E may play an important role in treating DR.

**Table 3:** The association between Vitamin Intake and DR.

Vitamin Intake	Model 1		Model 2		Model 3	
	Beta (95% CI)	P	Beta	P	Beta	P
Vit B6	-0.023 (-0.033, -0.013)	<0.00001	-0.019 (-0.029, -0.008)	0.00042	-0.017 (-0.027, -0.007)	0.0012
Vit E	-0.004 (-0.007, -0.002)	0.00032	-0.003 (-0.005, -0.001)	0.026	-0.002 (-0.005, -0.001)	0.039

Model 1: Non-adjusted.

Model 2: Adjust by age, sex, race.

Model 3: Adjust for: age, sex, race, education, bmi, vitamin A, C, K.

**Figure 7:**
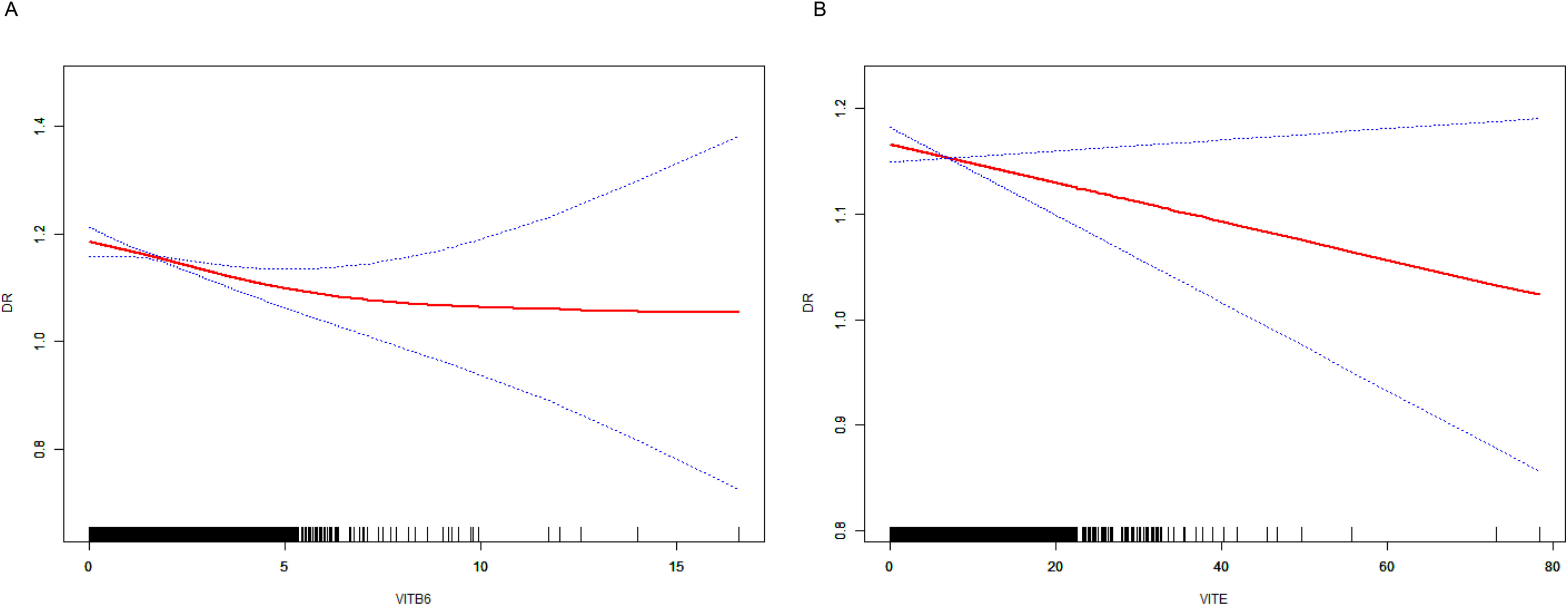
The result of the smooth curve of vitamins and DR. **(A)** the smooth curve of vitamin B6 and DR. **(B)** the smooth curve of vitamin E and DR.

We performed a smooth curve fit to describe the nonlinear relationship between vitamins and DR. Using a two-segment linear regression model, we found a nonlinear relationship between vitamin B6, E, and DR, suggesting that with increased intake of vitamin B6 (**Figure 7A**) and vitamin E (**Figure 7B**), there is a noticeable decrease in the risk and severity of DR.

## Discussion

In this study, we utilized MR analysis and cross-sectional data to explore the association between Cats, immune cells, metabolites, vitamins, and DR. Our findings indicate the pathogenic effects of CAT H on DR and the protective effects of vitamin B6, and E. Additionally, we highlighted the causal effects of immune cells, metabolites in DR, and the interactions between immune cells and CAT H. This study is pioneering in combining MR analysis with a cross-sectional study based on the NHANES database, systematically analyzing the causal relationships and correlations between various exposures and DR. By corroborating findings from both approaches, we have enhanced the reliability of our results, suggesting that CAT H could become crucial indicators of the risk of DR, and vitamin B6 and E could become potential therapeutics for DR.

The development and progression of diabetes and diabetic complications involve a highly complex process in which proteolytic events play a crucial role (42–45). Among these, CATs have garnered significant interest for their role in proteolytic events. In this study, we conducted a large-scale genetic-based MR analysis to investigate the causal relationship between nine different CATs and the risk and the progression of DR. Through two-sample and mediation MR analyses, we identified CAT H as a significant risk factor for DR. Additionally, we found that immune cells can influence DR mediated by CAT H. Some studies indicate that patients with the type 1 diabetes mellitus (T1DM) risk variant often exhibit higher CAT H transcription levels, an earlier onset of the disease, and a rapid decline in β-cell function (46, 47). CAT H, a lysosomal cysteine protease, plays a prominent role in physiological and pathological processes due to its unique endopeptidase activity (25). We have identified a significant positive causal relationship between CAT H and both NPDR and PDR, suggesting that CAT H may play a significant role in the onset and progression of DR. Previous research found that CAT B, L, and H are expressed constitutively in most immune cells, playing roles in both innate and adaptive immune responses (48). Cathepsin H is a lysosomal cysteine protease involved in protein degradation and processing. In the context of diabetic retinopathy, cathepsins have been implicated in the remodeling of the extracellular matrix (ECM) and in the regulation of inflammation (49). Dysregulation of cathepsin H can contribute to excessive ECM degradation, leading to retinal damage and the progression of DR. Additionally, cathepsin H may influence the activation of various signaling pathways that are involved in inflammatory responses, further exacerbating retinal damage. Previous studies have shown that increased cathepsin activity is associated with higher levels of inflammation and tissue degradation in diabetic complications (50), suggesting that cathepsin H could play a role in the pathogenesis of DR. In our study, we found that certain immune cells can increase the risk of DR by upregulating CAT H expression. This suggests that the interaction between immune cells and CAT H plays an important role in the pathogenesis of DR. Future research should conduct more experiments to investigate this further.

Recent studies have revealed that the pathological processes of DR are associated with long-term metabolic disturbances (51, 52). With advancements in metabolomics, metabolites and metabolic pathways linked to DR are continually being discovered (53). In our study, we utilized serum metabolomics GWAS to analyze NPDR and PDR, identifying metabolites causally linked to DR and subsequently performing enrichment analysis to determine the metabolic pathways most strongly associated with NPDR and PDR. Previous study found that sphingolipid metabolism may play an important role in DR pathogenesis (54), and another study suggested that caffeine metabolism will reduce the risk of DR in T2DM (55), the results of these studies are consistent with our findings. Previous research has identified the arginine metabolic pathway as a potential new treatment strategy for DR (56). Our study confirms that this pathway is significantly associated with DR, particularly in PDR, suggesting its crucial role in the progression of DR, especially in its later stages. Targeting this pathway may help reduce the incidence of PDR. In addition, we also found that the biosynthesis of valine, leucine, and isoleucine is highly significant in PDR, consistent with previous research findings (57). Based on our results, future research should focus on these pathways as potential drug targets, providing new strategies for the treatment and prevention of PDR.

Previous studies have found that vitamins may play an important role in DR (58, 59). A study found no significant differences in serum levels of vitamins B1 and B2 between diabetic patients and healthy controls, while levels of vitamins B6, B9, and B12 were significantly lower in diabetics. Additionally, only vitamin B12 levels were significantly lower in diabetic patients with retinopathy compared to those without, with no significant differences in B1, B2, B6, and B9 levels (60). Vitamin E, comprising tocopherol and tocotrienol, is recognized as one of the most potent antioxidants, known to suppress angiogenesis and reduce oxidative stress (61, 62). A Study also found that vitamin D levels in patients with DR are significantly lower than in control groups, indicating that vitamin D deficiency is a major risk factor for DR (63). Several studies have demonstrated that the mean serum level of vitamin E is significantly lower in patients with DR compared to those without DR (64, 65), but a study showed no significant difference in the serum vitamin E levels between patients with no DR and any DR severities (66). Despite previous studies exploring the relationship between vitamins and DR, the results remain controversial and lack large-scale confirmation. Through combined MR and NHANES analysis, we demonstrated a significant causal relationship between vitamins B6 and E and PDR, suggesting their important protective role in PDR. In our cross-sectional study, we found that blood levels of vitamin B6 progressively decreased across the non-DR, mild and moderate/severe NPDR, and PDR groups. Similarly, the intake levels of vitamins B1, B2, B6, B12, and E also decreased progressively. Along with MR analysis, these findings strongly suggest that these vitamins are directly related to the severity of DR and could serve as therapeutic targets for the disease. Further experiments are needed to validate the mechanisms through which vitamins affect DR. Additionally, our mediation MR analysis revealed that vitamin B6 could reduce the risk of PDR by acting on CAT H. Previous study found that vitamin B6 can inhibit the activity of CAT B, but its relationship with CAT H requires further experimental validation (67). Vitamin B6 helps reduce homocysteine levels and possesses anti-inflammatory properties, which may protect against vascular damage and microvascular complications in diabetic retinopathy (68). Vitamin E acts as an antioxidant, stabilizing cell membranes and reducing oxidative stress, which may protect retinal blood vessels and lower the risk of diabetic retinopathy (69). However, further experimental studies are needed to explore the biological mechanisms of Vitamin B6 and E in DR.

This study is the first to indicate that CAT H may have a causal effect on the risk and progression of DR. MR analysis effectively acts as a natural randomized controlled trial (RCT), and the GWAS data used were extracted from the latest versions, covering the largest European population to date. Therefore, the MR analysis method used in this study remains the most effective approach for determining the causal relationship between CATs and DR. Our study is the first to use mediation MR to investigate the relationship between immune cells and CATs, and further identify the mediating role of CAT H between immune cells and DR. Subsequently, we combined MR analysis and cross-sectional studies to identify the significant protective roles of vitamins B6 and E in DR, particularly in PDR. These findings suggest that the B vitamin family and vitamin E could serve as effective therapeutic targets for DR, especially in the treatment and prevention of PDR. Our findings provide constructive advice for the management of patients with DR, suggesting that the use of vitamin B6 and E in DR patients should be effective in preventing and treating PDR.

### Study limitations

The current study has several limitations: (1). As this study exclusively used GWAS data from the European population, it may not accurately represent other ethnicities or races worldwide. (2) In the cross-sectional study, despite our efforts to include a broad range of participants from NHANES, the number of participants was still limited, especially for the PDR group only has 31 patients, potentially leading to bias in the results. (3) Some covariates may not have been considered in the multiple regression analysis. (4) Despite efforts to identify and eliminate outlier variants, the potential for horizontal pleiotropy to affect the findings cannot be entirely ruled out. (5) A significant limitation of our study is the reliance on GWAS data predominantly derived from European populations and the use of NHANES data, which includes limited sample sizes for certain DR categories, particularly PDR. This reliance on a specific ethnic group limits the generalizability of our findings to other populations. The small sample size for PDR in the NHANES data may also introduce bias and affect the robustness of our results. Therefore, our findings should be interpreted with caution, and future research should aim to validate these associations in larger, more diverse populations. This approach will help ensure that the results are applicable across different ethnicities and enhance the overall reliability of the study. Therefore, further validation of this study’s findings is necessary through multi-center epidemiological research and genetic engineering experiments, employing larger sample sizes and diverse populations.

## Conclusion

Using MR analysis, this study was the first to explore the causal impact of CATs, immune cells, metabolites, and vitamins on DR from a genetic perspective, with findings validated by a cross-sectional study based on NHANES data. The results confirmed a causal association between CAT H and a significantly increased risk of DR, identified the metabolomic pathways that play an important role in the progression of DR and also explored the protective effects and therapeutic potential of vitamin B6 and E in PDR. However, the accuracy and validity of these findings require further verification through additional basic and clinical studies on DR.

## Data Availability

The original contributions presented in the study are included in the article/**Supplementary Material**. Further inquiries can be directed to the corresponding author.

## Supplementary material

Supplementary Figure 1 the MR result of immune cells and NPDR.

Supplementary Figure 2 the MR result of immune cells and PDR.
