# Supplementary figures and images for: Causal effect of immune cells, metabolites, cathepsins, and vitamin therapy in diabetic retinopathy: a Mendelian randomization and cross-sectional study
Source: Front Immunol. 2024 Oct 1;15:1443236. doi: 10.3389/fimmu.2024.1443236 (PMC11487118; doi:10.3389/fimmu.2024.1443236)

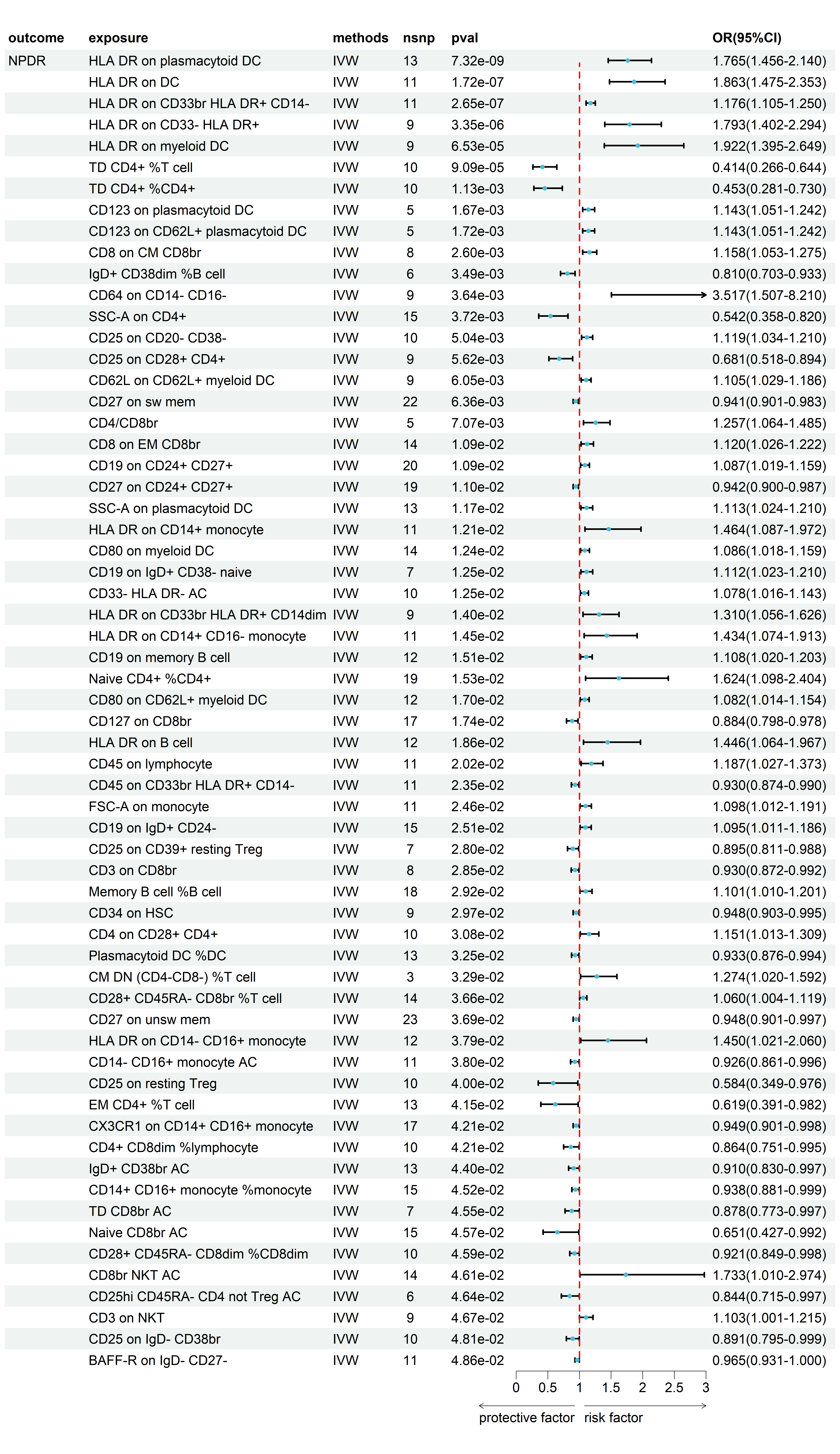

Supplement: Supplementary Figure 1 — the MR result of immune cells and NPDR. [file Image1.png]

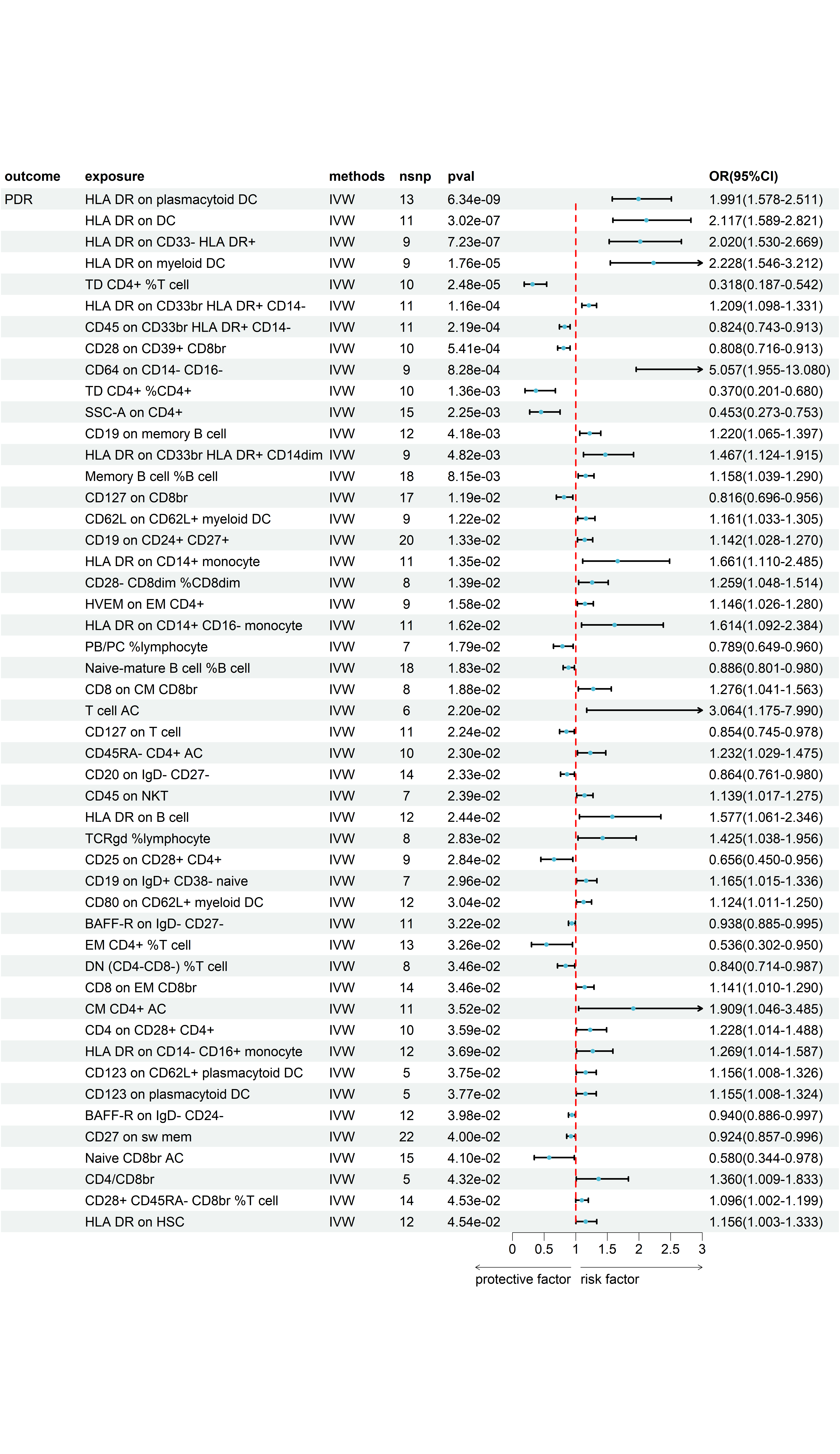

Supplement: Supplementary Figure 2 — the MR result of immune cells and PDR. [file Image2.png]
